# Effects of Lysine–Lysine Dipeptide on Serum Amino Acid Profiles, Intestinal Morphology, and Microbiome in Suckling Piglets

**DOI:** 10.3389/fnut.2022.881371

**Published:** 2022-05-10

**Authors:** Yuankun Deng, Hui Han, Liuqin He, Dun Deng, Jing Wang, Yulong Yin, Tiejun Li

**Affiliations:** ^1^College of Animal Science and Technology, Hunan Agricultural University, Changsha, China; ^2^Hunan Provincial Engineering Research Centre for Healthy Livestock and Poultry Production, Scientific Observing and Experimental Station of Animal Nutrition and Feed Science in South-Central, Ministry of Agriculture, Hunan Provincial Key Laboratory of Animal Nutritional Physiology and Metabolic Process, Key Laboratory of Agro-Ecological Processes in Subtropical Region, Institute of Subtropical Agriculture, Chinese Academy of Sciences, Changsha, China; ^3^Chinese Academy of Sciences, University of Chinese Academy of Sciences, Beijing, China; ^4^Tang Ren Shen Group, Zhuzhou, China; ^5^National Engineering Laboratory for Pollution Control and Waste Utilization in Livestock and Poultry Production, Changsha, China

**Keywords:** Lys-Lys dipeptide, amino acids, intestinal development, microbiota, suckling piglets

## Abstract

**Aims:**

Small peptides are more energy-saving and efficiently absorbed compared to amino acids. Our study aimed to evaluate the effect of the Lys-Lys dipeptide on the improvement of growth performance, amino acid metabolism, and gut development in suckling piglets.

**Methods and Results:**

Twenty-eight newborn suckling piglets were orally administrated with 0.1%, 1%, and 5% Lys-Lys dipeptide for 21 days. Our results showed that the Lys-Lys dipeptide has no significant effect on growth performance and intestinal morphology compared with the control group. We also found that the 1% Lys-Lys dipeptide significantly increased the concentrations of serum Lys, Thr, Phe, and Pro while decreasing Cys compared to the control group. Similarly, the 5% Lys-Lys dipeptide markedly increased the concentrations of serum Lys, Iso, Thr, Asp, Glu, and Pro compared to the control group. Moreover, the Lys-Lys dipeptide downregulated the expression of jejunal *Slc7a1, Slc7a2*, and *Slc15a1* and ileal *Slc7a2*. Additionally, the Lys-Lys dipeptide decreased the microbiota richness indices and relative abundance of *Bacteroidales*.

**Conclusion:**

In this study, we found that the Lys-Lys dipeptide contributes to the metabolism of amino acids but failed to affect the growth performance of piglets. Additionally, the Lys-Lys dipeptide decreased the relative abundance of *Bacteroidales*. These results provide a theoretical for the future application and research of Lys-Lys dipeptide in intestinal development of suckling piglets.

## Introduction

Amino acids play a key role in regulating various metabolic pathways such as the synthesis of proteins and peptides, glucose metabolism, and lipid metabolism, which are essential for the development and health of animals (1–6). Among these amino acids, lysine (Lys) is the first limiting amino acid for piglets. Previous studies have suggested that Lys restriction affected inflammatory status, muscle biochemical characteristics, and intestinal microbiota in piglets (7–9). Meanwhile, we also found that dietary Lys influenced the intestinal morphology and absorption of amino acids in piglets and mice (10, 11).

Small peptides, such as di- and tripeptides, are absorbed by the peptide transporter family (12). The transmembrane transport of short-chain peptides occurs in all living organisms and provides an efficient and energy-saving route for the uptake of bulk quantities of amino acids in the peptide form while providing an efficient and energy-saving source of their corresponding amino acids (13). Recently, several studies have been conducted to estimate the advantages of using small peptides (14). For example, administration of the alanyl-glutamine dipeptide increased weight gain in calves (15). Phenylalanine-cysteine exerted antioxidative effects (16), and lysine-glutamate displayed antitumor activity (17). Alanyl-glutamine increased glutamine levels in plasma and muscles and had anti-inflammatory effects (18). The glycyl-glutamine dipeptide has been reported to improve glutamine catabolism and jejunal cell proliferation and inhibit jejunal cell apoptosis (19). Glycyl-glutamine had beneficial effects on the composition of gut microbiota of piglets by increased bacterial loading, elevated alpha diversity, and increased proportions of anaerobes and fiber-degrading bacteria (20).

In our previous study, we have found that the Lys-Lys dipeptide increased cellular Pept1 abundance and affected the expression of amino acid transporters (such as *Slc7a1, Slc3a1*, and *Slc7a9*) and Lys concentration in IPEC-J2 (10). Meanwhile, the Lys-Lys dipeptide had an influence on the metabolism of amino acids and alleviated Lys deficiency-induced jejunal and ileal apoptosis in mice (10). Thus, we supposed that dietary supplementation with the Lys-Lys dipeptide may exert a beneficial effect on growth performance in suckling piglets. Here, we determined the potential effects of the Lys-Lys dipeptide on growth performance, intestinal development, and microbiota in suckling piglets.

## Materials and Methods

### Animals and Experimental Design

This study was conducted in accordance with the guidelines of the Institute of Subtropical Agriculture, Chinese Academy of Sciences. All experimental protocols were approved by the animal ethical committee of the Institute of Subtropical Agriculture, Chinese Academy of Sciences. Twenty-eight piglets were randomly divided into 4 groups after birth (*n* = 7/group): a) the control group in which piglets received 5 ml drinking water; b) the 0.1% Lys-Lys dipeptide group in which piglets received 5 ml Lys-Lys dipeptide solution (0.1 g dissolved in a final volume of 99.9 ml, oral administration); c) the 1% Lys-Lys dipeptide group in which piglets received 5 ml Lys-Lys dipeptide solution (1 g dissolved in a final volume of 99 ml, oral administration); d) the 5% Lys-Lys dipeptide group in which piglets received 5 ml Lys-Lys dipeptide solution (5 g dissolved in a final volume of 95 ml, oral administration). The piglets were housed with sows, free to breast milk, and received water or the Lys-Lys dipeptide on days 3, 5, 7, 14, and 21 after birth.

### Growth Performance and Blood Biochemical Parameters

The body weights of the piglets were monitored daily during the whole experimental period. Blood samples from overnight-fasted piglets were collected in plastic uncoated tubes, and serum was obtained by centrifugation at 3, 000 rpm for 10 min at 4°C and stored at −20°C until analysis according to previous studies (2, 21, 22). After blood sampling, the piglets were anesthetized with Zoletil 50 and killed for sample collection. Then, relative organ weights were calculated: organ/body weight.

### Intestinal Morphology Examination

Middle ileal sections were fixed with 4% paraformaldehyde-PBS overnight, and then dehydrated and embedded in paraffin blocks. After that, sections of 5 μm were cut and mounted on slides. The sections were further deparaffinized and hydrated and then stained with hematoxylin and eosin (H&E). Villus length and crypt depth were determined using the Image J software.

### Amino Acid Determination

A total of 0.5 ml of serum was centrifuged at 3,000 rpm for 5 min, and then supernatants were mixed with equivalent 10% sulfosalicylic acid for 1 h at 4°C. The mixtures were further centrifuged at 10,000 rpm for 15 min. After that, the supernatants were used to analyze amino acid contents with high-speed amino acid analyzer L-8900 (Japan).

### Real-Time Quantitative PCR

Total RNA from the jejunum and ileum samples was isolated from liquid nitrogen using the TRIZOL reagent (Invitrogen, United States). RNA concentration was measured with the Nanodrop one (Thermo Fisher, United States) and then treated with DNase I (Invitrogen, United States) according to the instruction of the manufacturer. Synthesis of first-strand cDNA was performed using PrimeScript Enzyme Mix 1, RT Primer Mix, and 5 × PrimerScript Buffer 2. Reverse transcription was conducted at 37°C for 15 min and at 85°C for 5 s. The primers (Table 1) used in this study were presented in the previous study (7). β-actin was chosen as a housekeeping gene to normalize target gene transcript levels. Real-time PCR was performed according to the manufacturer's instruction (LightCyclerR480II, Roche, Switzerland). Briefly, 1-μl of cDNA template was added to a total volume of 10 μl containing 5 μl of SYBR Premix Ex Taq II, 0.4 μl of PCR forward primer, 0.4 μl of PCR reverse primer, 0.2 μl of ROX reference dye, and 3 μl of ddH_2_O. We used the following protocol: (i) pre-denaturation programmer (30 s at 95°C); (ii) an amplification and quantification program consisting of repeated 40 cycles (5 s at 95°C and 31 s at 60°C); (iii) a melting curve program (15 s at 95°C, 1 min at 60 °C, and 15 s at 95 °C). Relative expression was expressed as a ratio of the target gene to the control gene using the formula 2^−(ΔΔCt)^, where ΔΔCt = (Ct_Target_-Ct_β−*actin*_) treatment-(Ct_Target_-Ct_β−*actin*_) control. Relative expression was normalized and expressed relative to the expression in the control group.

**Table 1:** Primers used for quantitative reverse transcription PCR.

**Gene**	**Accession no**.	**Sequence (5^**′**^-3^**′**^)**
*β-actin*	XM_003124280.4	F: CTGCGGCATCCACGAAACT R: AGGGCCGTGATCTCCTTCTG
*Slc7a1*	NM_001012613.1	F: TCTGGTCCTGGGCTTCATAA R:ACCTTCGTGGCATTGTTCAG
*Slc7a2*	NM_001110420.1	F: GCAACAACTGGCGAAGAAGT R: GGCATCATAAGGGTCAAAGC
*Slc15a1*	NM_214347	F: CAGACTTCGACCACAACGGA R: TTATCCCGCCAGTACCCAGA

### Intestinal Microbiome Analysis

Total genome DNA was extracted from colonic contents, and the bacterial 16S rRNA gene was amplified using the 341F/806R primer set targeting the V3-V4 region (341F, 5′-CCTAYGGGRBGCASCAG-3′; 806R, 5′-GGACTACNNGGGTATCTAAT-3′). Amplicons were extracted from 2% agarose gel and purified using a GeneJET gel extraction kit (Thermo Scientific) according to the manufacturer's instruction. Then, fragment libraries were prepared using the Ion Plus Fragment Library Kit 48 rxns (Thermo Fisher). After quantification and purification, the amplicons were sequenced.

The sequences were analyzed and assigned to operational taxonomic units (OTUs; 97% identity). Then, the representative sequences were selected using the Uparse software package. Chao 1, ACE, Shannon, and Simpson indices were calculated to analyze alpha-diversity, while principal component analysis (PCA) and principal coordinate analysis (PCoA) were performed to analyze beta-diversity. Raw reads were deposited in the NCBI Sequence Read Archive (SRA) database (accession number: PRJNA813170).

### Statistical Analysis

All data were analyzed between two groups by using Student's *t*-test (SPSS 22.0 software). The data are expressed as the mean ± SEM. Differences of *P* < 0.05 were considered significant.

## Results

### Effect of the Lys-Lys Dipeptide on Growth Performance

**Figure 1:**
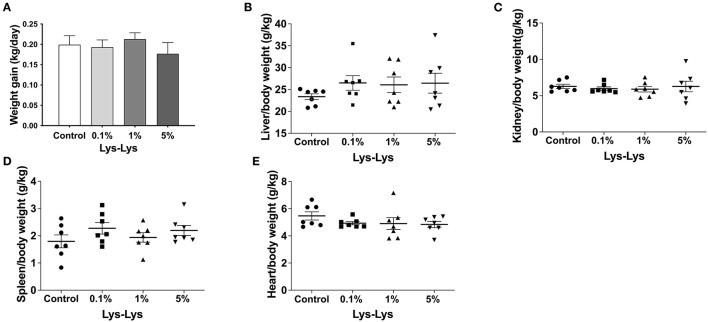
Effect of the Lys-Lys dipeptide on growth performance in piglets. **(A)** Weight gain and **(B–E)** organ relative weight were calculated according to the formula organ/body weight. Data were expressed as the mean ± SEM (*n* = 7).

Compared with the control group, the 1% Lys-Lys dipeptide tended to increase weight gain and the 5% Lys-Lys dipeptide tended to decrease the weight gain of the piglets, but the effects were insignificant (Figure 1A). Meanwhile, the Lys-Lys dipeptide had little effect on the weights of the liver, kidney, and spleen, and heart relative weight (Figures 1B–E).

### Effect of the Lys-Lys Dipeptide on Intestinal Morphology

**Figure 2:**
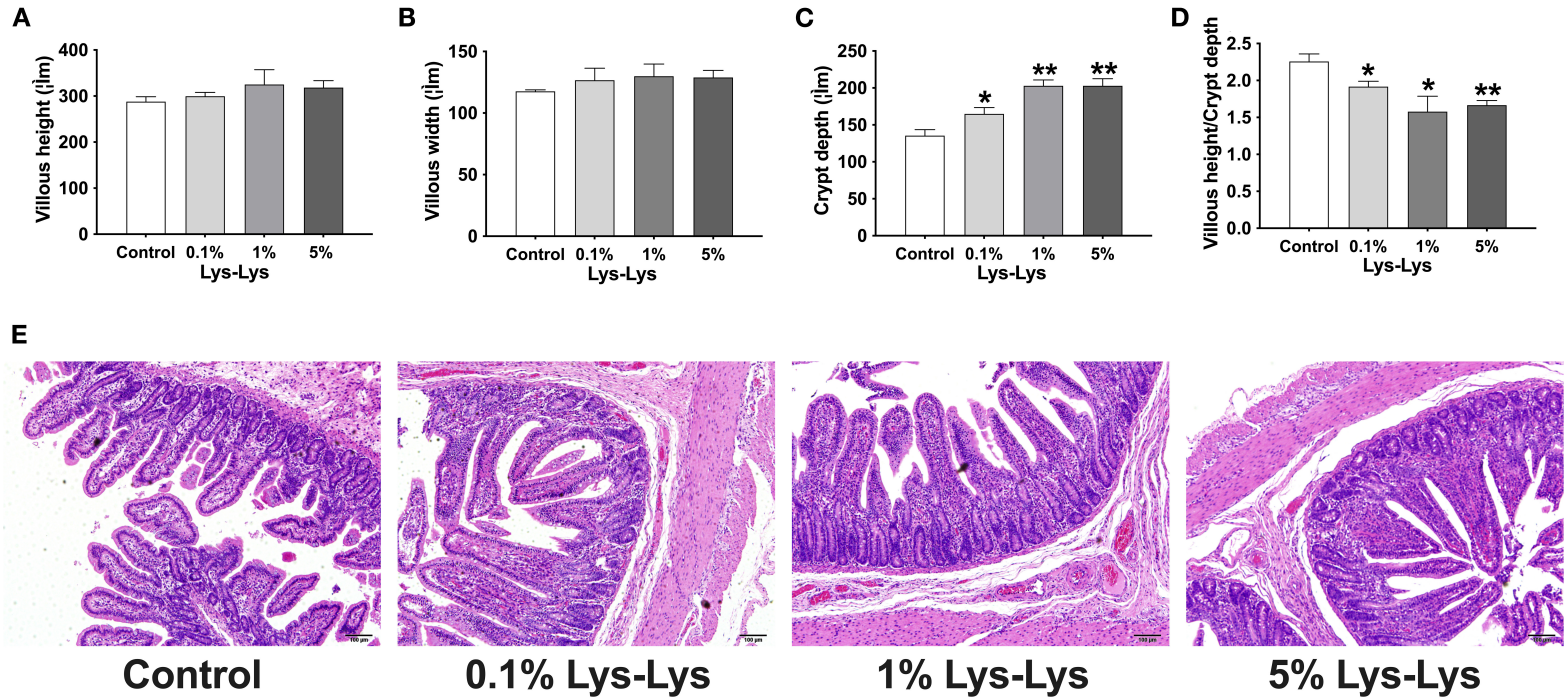
Effect of the Lys-Lys dipeptide on morphological characteristic of the ileum in piglets. **(A)** Villous height, **(B)** villous width, **(C)** crypt depth, and **(D)** villous height/crypt depth. **(E)** H&E staining of the ileum in piglets. Scale bar = 100 μm. Data were expressed as the mean ± SEM (*n* = 7). *Different from control, *P* < 0.05; **different from control, *P* < 0.01.

As shown in Figure 2, Lys-Lys dipeptide supplementation tended to increase villous height and villous width, resulting in increased villous surface area, compared to the control group (*P* > 0.05). The 0.1% (*P* < 0.05), 1% (*P* < 0.01), and 5% (*P* < 0.01) Lys-Lys dipeptide treatments significantly increased crypt depth compared with the control group. Meanwhile, the 0.1% (*P* < 0.05), 1% (*P* < 0.05), and 5% (*P* < 0.01) dipeptide supplementations markedly decreased the ratio of villous height to crypt depth.

### Effect of the Lys-Lys Dipeptide on Blood Biochemical Parameters

We detected the content of serum-related enzyme, immune indices, and metabolic by-products in piglets serum. The results showed that the Lys-Lys dipeptide had little effect on serum-related enzymes (ALP, ACP, γ-GT, α-AMS, and Amy-p) and immune indexes (TP, ALB, IgG, and IgM) of the piglets (Table 2). However, the serum concentrations of LDL and CHOL in the 1% Lys-Lys and 5% Lys-Lys groups were significantly lower than those in the control group (*P* < 0.01). Also, compared to the control group, the 1% Lys-Lys dipeptide significantly decreased the serum concentration of Mg (*P* < 0.05). The 0.1% Lys-Lys dipeptide had no significant effects on serum biochemical parameters.

**Table 2:** Effect of Lys-Lys on blood biochemical parameters of piglets^a^.

**Item**	**Control**	**0.1% Lys-Lys**	**1% Lys-Lys**	**5% Lys-Lys**
ALP, U/L	184.29 ± 28.07	182.57 ± 26.57	150.80 ± 5.49	236.33 ± 33.64
ACP, U/L	17.12 ± 2.26	20.08 ± 3.87	17.88 ± 2.04	19.11 ± 4.10
γ-GT, U/L	80.17 ± 14.81	62.50 ± 8.20	64.86 ± 12.05	81.00 ± 16.32
α-AMS, U/L	2017.14 ± 307.64	2143.71 ± 289.39	1967.14 ± 274.77	1783.33 ± 244.09
Amy-p	2027.14 ± 303.40	2134.14 ± 278.34	1980.16 ± 274.23	1804.50 ± 245.90
TP, g/L	48.34 ± 0.43	47.34 ± 2.29	46.27 ± 1.69	47.08 ± 1.93
ALB, g/L	33.21 ± 2.11	31.79 ± 1.65	33.21 ± 2.05	31.00 ± 0.96
IgG, g/L	1.26 ± 0.17	1.19 ± 0.10	1.04 ± 0.04	0.98 ± 0.09
IgM, g/L	0.31 ± 0.03	0.28 ± 0.02	0.26 ± 0.02	0.33 ± 0.05
LACT, mmol/L	9.97 ± 1.14	10.01 ± 1.08	9.60 ± 0.66	9.61 ± 0.65
NH3L, μmol/L	816.86 ± 78.01	703.79 ± 77.75	647.97 ± 44.84	683.92 ± 51.18
UREA, mmol/L	3.60 ± 0.74	3.51 ± 0.43	3.77 ± 0.41	4.00 ± 0.48
GLU, mmol/L	2.24 ± 0.60	2.77 ± 0.52	3.17 ± 0.45	2.10 ± 0.14
CHOL, mmol/L	4.09 ± 0.20	3.75 ± 0.30	3.25 ± 0.12**	2.99 ± 0.17**
TG, mmol/L	0.73 ± 0.11	0.73 ± 0.08	0.52 ± 0.03	0.74 ± 0.09
HDL, mmol/L	1.71 ± 0.08	1.79 ± 0.07	1.54 ± 0.07	1.53 ± 0.07
LDL, mmol/L	2.51 ± 0.21	2.21 ± 0.42	1.78 ± 0.12**	1.61 ± 0.15**
Ca, mmol/L	2.42 ± 0.12	2.46 ± 0.14	2.41 ± 0.13	2.32 ± 0.11
P, mmol/L	5.30 ± 0.44	4.81 ± 0.32	4.42 ± 0.26	4.96 ± 0.29
Mg, mmol/L	1.63 ± 0.13	1.56 ± 0.05	1.25 ± 0.07**	1.39 ± 0.12
Ironl, μmol/L	16.53 ± 1.43	21.10 ± 1.60	21.02 ± 1.44	17.33 ± 2.10

a *Data were expressed as the mean ± SEM (n = 7)*.

** *Different from control, P < 0.01*.

### Effect of the Lys-Lys Dipeptide on Serum Amino Acids

The 1% Lys-Lys dipeptide treatment significantly increased serum concentrations of Lys (*P* < 0.05), threonine (*P* < 0.05), phenylalanine (*P* < 0.05), and proline (*P* < 0.01) but markedly decreased that of cysteine (*P* < 0.01) compared to the control group (Table 3). Meanwhile, the serum concentrations of Lys (*P* < 0.05), isoleucine (*P* < 0.05), threonine (*P* < 0.01), aspartate (*P* < 0.05), glutamate (*P* < 0.05), and proline (*P* < 0.05) were markedly higher in the 5% Lys-Lys dipeptide group compared with the control group. The 0.1% Lys-Lys dipeptide had no significant effects on serum amino acids (Table 3).

**Table 3:** Effect of the Lys-Lys dipeptide on serum amino acids in piglets^a^.

**Item**	**Control**	**0.1% Lys-Lys**	**1% Lys-Lys**	**5% Lys-Lys**
Lysine	44.96 ± 2.74	49.08 ± 0.16	80.57 ± 3.80*	63.10 ± 5.10*
Methionine	11.86 ± 0.83	13.52 ± 1.70	14.69 ± 2.18	12.80 ± 1.33
Valine	42.20 ± 4.64	40.34 ± 4.37	53.11 ± 7.27	48.59 ± 3.86
Isoleucine	26.63 ± 2.39	26.88 ± 1.74	29.20 ± 3.12	34.71 ± 1.75*
Leucine	34.25 ± 3.83	33.22 ± 1.99	45.76 ± 3.78	41.18 ± 3.53
Threonine	19.96 ± 1.23	22.64 ± 2.81	30.65 ± 3.95*	31.72 ± 3.00**
Tryptophan	7.91 ± 0.47	7.73 ± 0.54	10.10 ± 1.06	8.63 ± 0.70
Phenylalanine	19.87 ± 1.30	19.49 ± 1.17	25.54 ± 2.02*	23.02 ± 1.85
Histone	14.33 ± 1.66	15.11 ± 1.47	19.52 ± 2.36	13.35 ± 3.93
Serine	22.52 ± 1.86	22.29 ± 1.68	29.22 ± 3.49	24.96 ± 1.91
Arginine	22.78 ± 2.60	19.77 ± 1.84	30.08 ± 3.53	25.77 ± 3.61
Glycine	77.64 ± 12.13	80.08 ± 6.83	99.34 ± 8.17	88.17 ± 8.65
Aspartate	4.54 ± 0.72	4.98 ± 0.70	5.98 ± 0.96	7.36 ± 1.02*
Glutamate	79.81 ± 11.44	92.65 ± 5.70	107.53 ± 9.83	110.52 ± 3.37*
Tyrosine	31.91 ± 1.11	30.14 ± 1.54	35.05 ± 2.40	35.27 ± 1.93
Proline	33.73 ± 1.91	40.80 ± 2.40	60.21 ± 2.97**	45.90 ± 3.16*
Cysteine	10.37 ± 0.99	8.99 ± 0.81	6.26 ± 0.70**	12.64 ± 0.92
Alanine	82.19 ± 8.74	85.69 ± 3.26	92.04 ± 9.50	89.23 ± 8.52

a Data were expressed as the mean ± SEM (n = 7). *Different from control, P < 0.05;

** *Different from control, P < 0.01*.

### Effect of the Lys-Lys Dipeptide on Amino Acid Transporters in the Intestine

**Figure 3:**
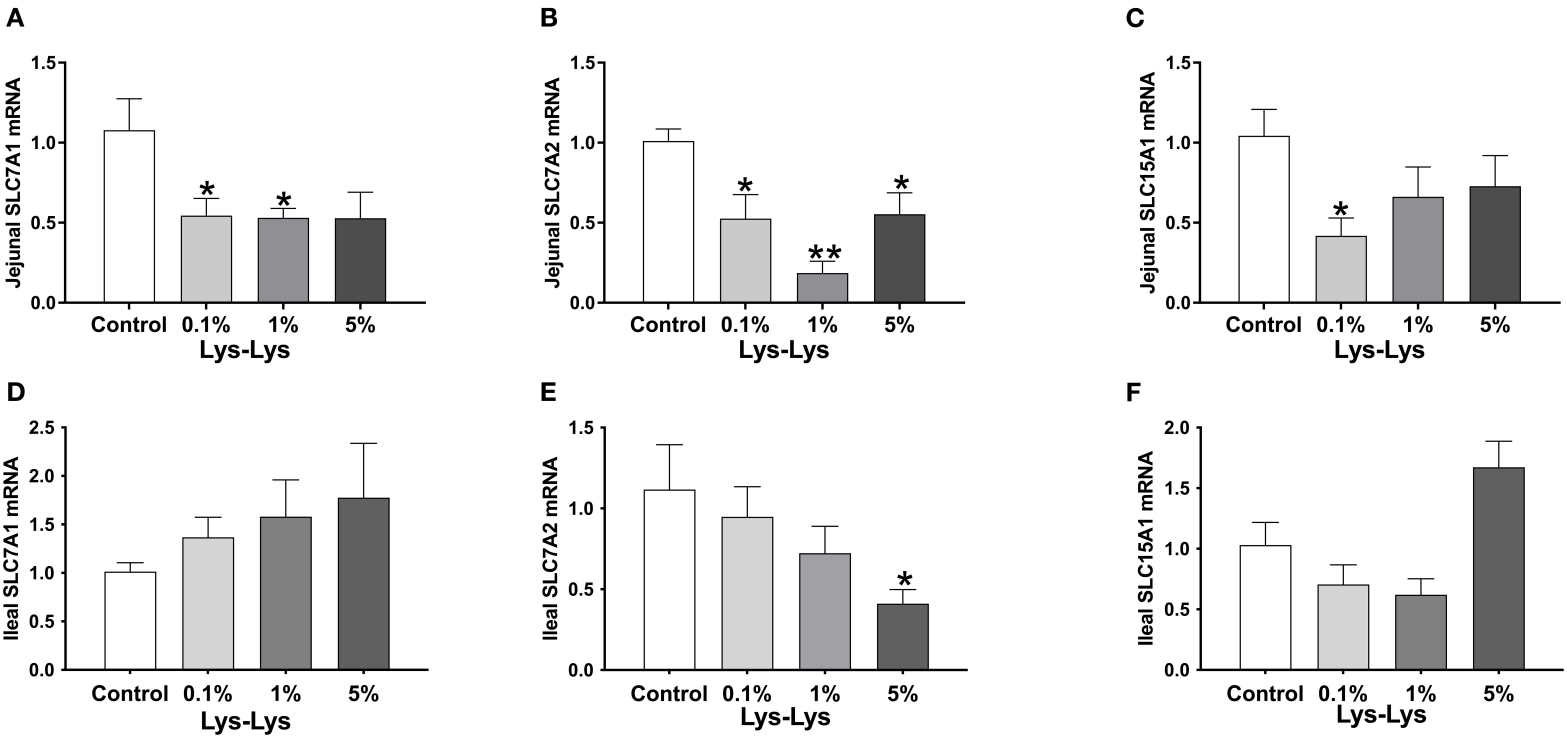
Effect of the Lys-Lys dipeptide on intestinal lysine and dipeptide transporters in piglets. **(A)** Jejunal *Slc7a1*. **(B)** Jejunal *Slc7a2*. **(C)** Jejunal *Slc15a1*. **(D)** Ileal *Slc7a1*. **(E)** Ileal *Slc7a2*. **(F)** Ileal *Slc15a1*. Data were expressed as the mean ± SEM (*n* = 7). *Different from control, *P* < 0.05; **different from control, *P* < 0.01.

In the jejunum, *Slc7a1* was significantly lower in the 0.1% and 1% Lys-Lys dipeptide groups (*P* < 0.05), and *Slc7a2* was markedly lower in all the Lys-Lys dipeptide-treated groups (*P* < 0.05) compared to the control group (Figures 3A,B). Meanwhile, 0.1% Lys-Lys dipeptide significantly inhibited the expression of *Slc15a1* (*P* < 0.05) (Figure 3C). 0.1%, 1% and 5% Lys-Lys dipeptide group *Slc7a1* had no significant difference (*P* > 0.05) compared to the control group (Figure 3D). In the ileum, the 5% Lys-Lys dipeptide markedly inhibited the expression of *Slc7a2* (*P* < 0.05) compared to the control group (Figure 3E). 0.1%, 1% and 5% Lys-Lys dipeptide group *Slc15a1* had no significant difference (*P* > 0.05) compared to the control group (Figure 3F).

### Effect of the Lys-Lys Dipeptide on Intestinal Microbiota

**Figure 4:**
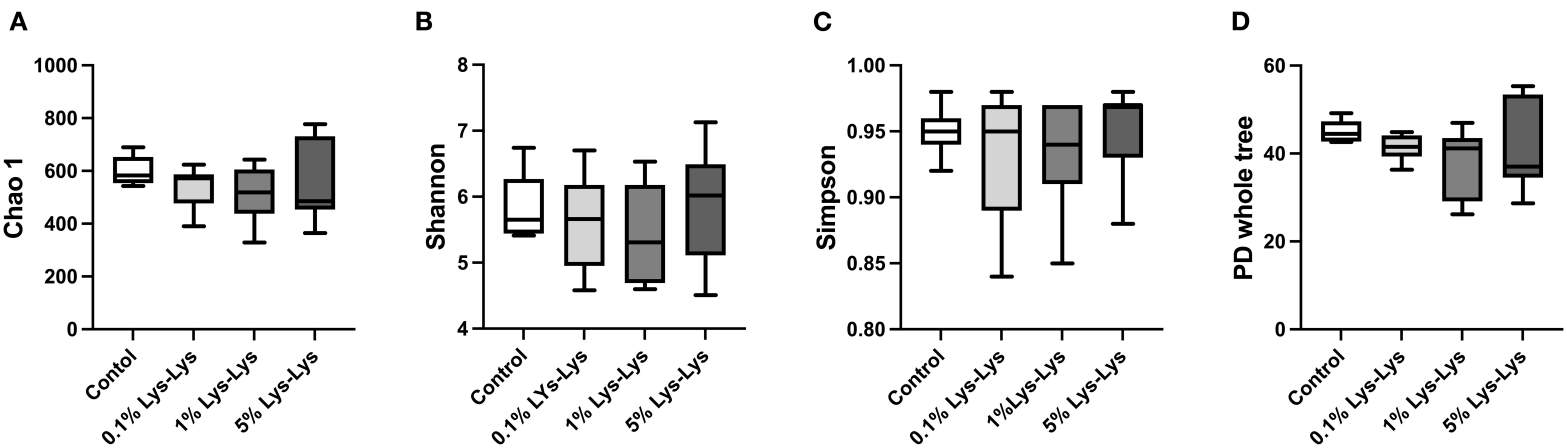
Effect of the Lys-Lys dipeptide on intestinal microbiota in piglets. Data were expressed as the mean ± SEM (*n* = 7). *Different from control, *P* < 0.05. **(A)** Chao1. **(B)** Shannon. **(C)** Simpson. **(D)** PD whole tree.

We tested the indexes of Chao 1, Shannon, Simpson, and PD whole tree. Chao 1 accounted for microbial community richness while Shannon and Simpson accounted for microbial community diversity. However, there were no differences in the index of Chao 1, Shannon, Simpson, and PD whole tree between the control group and the Lys-Lys dipeptide-supplemented groups (Figures 4A–D).

**Figure 5:**
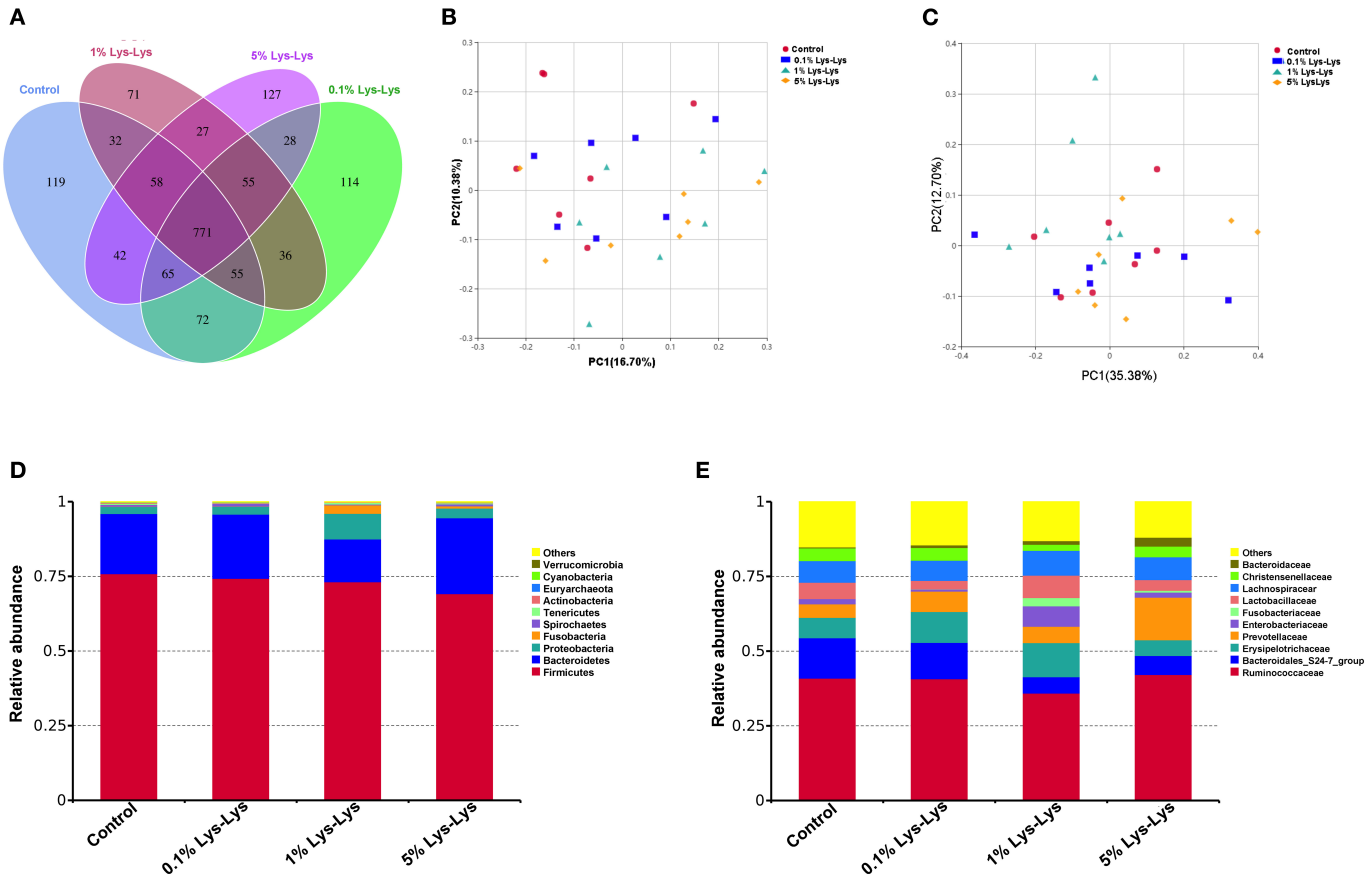
Effect of the Lys-Lys dipeptide on intestinal microbiota in piglets. **(A)** Venn diagram illustrating the overlap of operational taxonomic units (OTUs) in the intestinal microbiota of piglets. **(B)** Principal coordinate analysis (PCoA) of unweighted UniFrac distance. **(C)** PCoA of weighted UniFrac distance. **(D)** Relative abundance of the top 10 phyla in each sample. **(E)** Relative abundance of the top 10 families in each sample. Data were expressed as the mean ± SEM (*n* = 7). *Different from control, *P* < 0.05.

The Venn diagram showed that the control piglets and piglets fed with the Lys-Lys dipeptide contained 771 same OTUs (Figure 5A). The control and the 0.1%, 1%, and 5% Lys-Lys dipeptide groups contained 119, 114, 71, and 127 unique OTUs, respectively.

A principal coordinate analysis was used to analyze the clustering of microbiota within groups. In this study, PCoA of both unweighted and weighted UniFrac distances showed no clear separation between the control group and the Lys-Lys dipeptide-supplemented groups (Figures 5B,C).

At the phylum level, the relative abundance of *Bacteroidetes* was lower in the 1% Lys-Lys group than that in the control group (0.2 ± 0.04 vs. 0.14 ± 0.04), but the difference was insignificant (*P* > 0.05) (Table 4, Figure 5D). At the family level, we selected 10 microbiological families with relatively large differences for analysis. The 1% Lys-Lys dipeptide significantly decreased the relative abundance of *Bacteroidales* (0.13 ± 0.03 vs. 0.06 ± 0.02, *P* < 0.05) (Table 4, Figure 5E).

**Table 4:** Effect of Lys-Lys on bacteria at the phylum and family levels in piglets^a^.

**Item**	**Control**	**0.1% Lys-Lys**	**1% Lys-Lys**	**5% Lys-Lys**
**Phylum**				
Firmicutes	0.76 ± 0.04	0.74 ± 0.07	0.73 ± 0.06	0.69 ± 0.07
Bacteroidetes	0.20 ± 0.04	0.21 ± 0.07	0.14 ± 0.02	0.25 ± 0.07
Proteobacteria	0.02 ± 0.01	0.03 ± 0.01	0.08 ± 0.03	0.03 ± 0.01
**Family**				
Ruminococcaceae	0.41 ± 0.05	0.41 ± 0.05	0.36 ± 0.05	0.42 ± 0.08
Bacteroidales	0.13 ± 0.30	0.12 ± 0.06	0.06 ± 0.02*	0.06 ± 0.02
Erysipelotrichaceae	0.07 ± 0.03	0.10 ± 0.05	0.11 ± 0.05	0.05 ± 0.02
Prevotellaceae	0.05 ± 0.02	0.07 ± 0.03	0.05 ± 0.02	0.14 ± 0.06
Enterobacteriaceae	0.02 ± 0.01	0.01 ± 0.002	0.07 ± 0.03	0.02 ± 0.01

a *Data were expressed as the mean ± SEM (n = 7)*.

* *Different from control, P < 0.05*.

## Discussion

Dipeptides can be uptaken by the intestine and are involved in various molecular pathways including those for immune function, proliferation, apoptosis, and oxidative stress (16–19). In our current study, we found that the Lys-Lys dipeptide had no effect on the growth performance of suckling piglets, which is consistent with the research conducted by Nosworthy et al. (23). Meanwhile, previous studies have shown that lysine restriction increased intramuscular fat contents (9). This may be related to the growth performance of piglets, and the specific mechanism needs to be further studied. We suspect that Lys from sow milk was enough for suckling piglets to maintain growth. Moreover, sucking piglets might not have absorbed and utilized the Lys-Lys dipeptide. It is evident that the small intestine plays an important role in nutrition transport and absorption. Morphology structures of the small intestine are also regarded as indicators of reflecting intestinal development. For example, intestinal crypts host epithelial stem cells, which can differentiate into mature intestinal epithelial cells as they migrate up the villus (24, 25). Villous surface implies the intestinal absorptive capacity of nutrients (26). Our previous study has shown that Lys was involved in enhancing intestinal crypt height and villus depth and differentially affected intestinal cationic amino acid transporter expression (11, 27). However, our present results showed that Lys-Lys had no effect on villous height, indicating that Lys-Lys does not affect the intestinal absorptive capacity of suckling piglets. The results suggested that Lys-Lys may be involved in increasing the turnover rate of intestinal mucosal cells (28) and that it had little effect on the nutrition absorption of suckling piglets.

Dipeptides are reported to be involved in immune response (15). Previous reports have confirmed that peptides possess a high affinity to metal ions and increase the absorption of metal ions (29). However, in this study, the Lys-Lys dipeptide had little effect on the serum-related immune index in piglets, and Mg ion level was lower in piglets treated with the 1% Lys-Lys dipeptide than in the control group, suggesting that Lys-Lys promotes the absorption of Mg in piglets. We also analyzed the effect of the Lys-Lys dipeptide on serum-related enzyme and metabolic by-products, which indicated that Lys-Lys had no effect on serum-related enzyme and immune indexes. Since LDL and CHOL are involved in lipid metabolism (30, 31), the results from this study showed that the 1% and 5% Lys-Lys dipeptide decreased the serum LDL and CHOL levels compared with the control group. The study found that Lys also significantly decreased lipid accumulation in the liver and increased weight loss in rats (20). We suggest that the Lys-Lys dipeptide may participate in lipid metabolism. However, the potential mechanism with which the Lys-Lys dipeptide exerts influence on lipid metabolism needs further investigation. Various studies have shown that a dipeptide mediates the metabolism of amino acids (32). Our previous study has also shown that the Lys-Lys dipeptide affects intracellular histidine, proline, threonine, and tyrosine abundances and extracellular asparagine, cysteine, glutamine, methionine, histidine, phenylalanine, proline, serine, tryptophan, and tyrosine abundances in IPEC-J2 cells (10). Moreover, the Lys-Lys dipeptide altered serum aspartic acid and tryptophan levels in mice (10). These are consistent with our current results showing that the Lys-Lys dipeptide influenced the serum concentrations of Lys, isoleucine, threonine, phenylalanine, aspartate, glutamate, proline, and cysteine in piglets. However, Lys content was decreased in the 5% Lys-Lys dipeptide compared with the 1% Lys-Lys dipeptide. This may be related to the intestinal amino acid transportability of piglets. The Lys-Lys dipeptide inhibited the expressions of jejunal *Slc7a1* and *Slc7a2* and ileal *Slc7a2*. However, the 0.1% Lys-Lys dipeptide decreased the expression of jejunal *Slc15a1*. These results suggested that absorption of the Lys-Lys dipeptide may upregulate Lys uptake, which further decreases *Slc7a1* and *Slc7a2* to maintain Lys balance. *Slc15a1*, known as PepT1, is mainly expressed in the small intestine and participates in intestinal absorption of di-and tripeptides (33). We also found that the Lys-Lys dipeptide upregulated the expression of Pept1 in IPEC-J2 and that it inhibited Lys transporters (*Slc7a1* and *Slc7a2*) and increased the expression of Pept1 in mice (10). These results indicated that the Lys-Lys dipeptide affects the metabolism and transport of amino acids and that the effects vary according to different experimental models (cells, mice, and piglets).

Intestinal microbiota can be shaped by the consumption of various nutrients and modulate host health (34). Our previous study has shown that dietary Lys can affect intestinal microbiota. Once 100% dietary Lys changed to 70% Lys, intestinal microbial diversity was significantly increased. Long-term (6 weeks) dietary Lys restriction increased the abundances of *Actinobacteria, Saccharibacteria*, and *Synergistetes*. *Bacteroidales* have been shown to play a vital role in modulating host immune and intestinal functions (35, 36). In this study, we found that the Lys-Lys dipeptide decreased microbiota richness indices. The 0.1 and 5% Lys-Lys dipeptide has a significant effect on the relative abundance of bacteria compared to the control group. The 1% Lys-Lys dipeptide can decrease the relative abundance of the *Bacteroidales* family in piglets. However, Lys-Lys dipeptide had no significant difference at the level of microbial genus, and it had little effect on serum related immune indexes including TP, ALB, IgG, and IgM, suggesting that the Lys-Lys dipeptide did not induce an immune response in the piglets. Therefore, the mechanism of dipeptide-mediated intestinal microbiota on the intestinal barrier needs to be further explored.

In conclusion, we have found that the Lys-Lys dipeptide contributes to the metabolism of amino acids but failed to affect the growth performance of piglets. Additionally, the Lys-Lys dipeptide decreased the microbiota richness indices and relative abundance of Bacteroiditalics. The 1% Lys-Lys dipeptide was more forcefully effective than the other two doses. These results provide a theoretical basis for the intestinal development of suckling piglets, but the economic value of peptides in animal production needs further consideration.

## Data Availability Statement

The datasets presented in this study can be found in online repositories. The names of the repository/repositories and accession number(s) can be found at: https://www.ncbi.nlm.nih.gov/, PRJNA813170.

## Ethics Statement

The animal study was reviewed and approved by Institute of Subtropical Agriculture.

## Author Contributions

YD, HH, YY, and TL contributed to conception and design of the study. YD, HH, LH, and JW organized the database. YD, HH, JW, and DD performed the statistical analysis. YD, HH, and JW wrote the first draft of the manuscript. LH, DD, and TL wrote sections of the manuscript. All authors contributed to manuscript revision, read, and approved the submitted version.

## Funding

This study was supported by the Open Fund of Key Laboratory of Agro-ecological Processes in Subtropical Region, Chinese Academy of Sciences (ISA2018204), National Natural Science Foundation of China (31872371 and 31902168), Hunan Key Research Program (2017NK2321), Changsha Science and Technology Key Program (kq1703007 and kq1901090), Hunan High-Level Talent Gathering Project (2018RS3111), National Science Foundation for Outstanding Young Scholars of Hunan Province (2019JJ30017), and China Agriculture Research System (CARS-35). We are grateful to the Public Service Technology Center, Institute of Subtropical Agriculture, Chinese Academy of Sciences for the technical support.

## Conflict of Interest

DD was employed by Tang Ren Shen Group. The remaining authors declare that the research was conducted in the absence of any commercial or financial relationships that could be construed as a potential conflict of interest.

## Publisher's Note

All claims expressed in this article are solely those of the authors and do not necessarily represent those of their affiliated organizations, or those of the publisher, the editors and the reviewers. Any product that may be evaluated in this article, or claim that may be made by its manufacturer, is not guaranteed or endorsed by the publisher.

## References

[1] Wu G. Functional amino acids in nutrition and health. Amino Acids. (2013) 45:407–11. 10.1007/s00726-013-1500-6 23595206

[2] Yin J Ren W Huang X Li T Yin Y. Protein restriction and cancer. Biochim Biophys Acta. (2018) 1869:256–62. 10.1016/j.bbcan.2018.03.004 29596961

[3] Yin J Ren W Chen S Li Y Han H Gao J. Metabolic Regulation of Methionine Restriction in Diabetes. Mol Nutr Food Res. (2018) 62:e1700951. 10.1002/mnfr.201700951 29603632

[4] Yin J Conlon M Kim SW. Nutrients and inflammatory diseases. Mediators Inflamm. (2017) 2017:6134909. 10.1155/2017/6134909 28539705 PMC5429948

[5] Yin J Ren W Yang G Duan J Huang X Fang R. L-Cysteine metabolism and its nutritional implications. Mol Nutr Food Res. (2016) 60:134–46. 10.1002/mnfr.201500031 25929483

[6] Lv D Xiong X Yang H Wang M He Y Liu Y. Effect of dietary soy oil, glucose, and glutamine on growth performance, amino acid profile, blood profile, immunity, and antioxidant capacity in weaned piglets. Science China Life sciences. (2018) 61:1233–42. 10.1007/s11427-018-9301-y 29785573

[7] Yin J Han H Li Y Liu Z Zhao Y Fang R. Lysine Restriction Affects Feed Intake and Amino Acid Metabolism Via Gut Microbiome in Piglets. Cell Physiol Biochem. (2017) 44:1749–61. 10.1159/000485782 29216634

[8] Han H Yin J Wang B Huang X Yao J Zheng J. Effects of dietary lysine restriction on inflammatory responses in piglets. Sci Rep. (2018) 8:2451. 10.1038/s41598-018-20689-3 29402921 PMC5799382

[9] Palma-Granados P Haro A Seiquer I Lara L Aguilera JF Nieto R. Similar effects of lysine deficiency in muscle biochemical characteristics of fatty and lean piglets. J Anim Sci. (2017) 95:3025–36. 10.2527/jas.2017.1364 28727124

[10] Yin J Li Y Han H Zheng J Wang L Ren W. Effects of lysine deficiency and lys-lys dipeptide on cellular apoptosis and amino acids metabolism. Mol Nutr Food Res. (2017) 61:9. 10.1002/mnfr.201600754 28012236

[11] He L Yang H Hou Y Li T Fang J Zhou X. Effects of dietary L-lysine intake on the intestinal mucosa and expression of cat genes in weaned piglets. Amino Acids. (2013) 45:383–91. 10.1007/s00726-013-1514-0 23722415

[12] Smith DE Clemencon B Hediger MA. Proton-coupled oligopeptide transporter family Slc15: physiological, pharmacological and pathological implications. Mol Aspects Med. (2013) 34:323–36. 10.1016/j.mam.2012.11.003 23506874 PMC3602806

[13] Daniel H Kottra G. The proton oligopeptide cotransporter family Slc15 in physiology and pharmacology. Pflugers Archiv. (2004) 447:610–8. 10.1007/s00424-003-1101-4 12905028

[14] Li Y Zhao J Liu X Xia X Wang Y Zhou J. Transport of a novel angiotensin-I-converting enzyme inhibitory peptide Ala-His-Leu-Leu across human intestinal epithelial caco-2 cells. J Med Food. (2017) 20:243–50. 10.1089/jmf.2016.3842 28296590

[15] Zhou Y Zhang P Deng G Liu X Lu D. Improvements of immune status, intestinal integrity and gain performance in the early-weaned calves parenterally supplemented with L-Alanyl-L-glutamine dipeptide. Vet Immunol Immunopathol. (2012) 145:134–42. 10.1016/j.vetimm.2011.10.020 22100191

[16] Je J-Y Cho Y-S Gong M Udenigwe CC. Dipeptide Phe-Cys derived from in silico thermolysin-hydrolysed rubisco large subunit suppresses oxidative stress in cultured human hepatocytes. Food Chem. (2015) 171:287–91. 10.1016/j.foodchem.2014.09.022 25308671

[17] Chanyuan TAN Xin Z Yinfeng J Dongmei Z Junhui C. Antitumor activity of vilon dipeptide Lys-Glu. Chinese Pharmacological Bulletin. (2007) 23:233–6. 10.3321/j.issn:1001-1978.2007.02.024

[18] Maretta M Toth S Jonecova Z Vesela J. Impact of alanyl-glutamine dipeptide on proliferative and inflammatory changes in jejunal mucosa after acute mesenteric ischemia. J Pediatr Surg. (2014) 49:1385–9. 10.1016/j.jpedsurg.2014.01.056 25148743

[19] Wang H Jia G Chen Z-l Huang L Wu C-m Wang K-n. The effect of glycyl-glutamine dipeptide concentration on enzyme activity, cell proliferation and apoptosis of jejunal tissues from weaned piglets agricultural sciences in China. J Integr Agric. (2011) 10:1088–95. 10.1016/S1671-2927(11)60098-9

[20] Lin H-Y Chen C-C Chen Y-J Lin Y-Y Mersmann HJ Ding S-T. Enhanced amelioration of high-fat diet-induced fatty liver by docosahexaenoic acid and lysine supplementations. Biomed Res Int. (2014) 2014:310981. 10.1155/2014/310981 24967351 PMC4055637

[21] Cho BY Park MR Lee JH Ra MJ Han KC Kang IJ. Standardized cirsium setidens nakai ethanolic extract suppresses adipogenesis and regulates lipid metabolisms in 3t3-L1 adipocytes and C57bl/6j mice fed high-fat diets. J Med Food. (2017) 20:763–76. 10.1089/jmf.2017.3965 28686516

[22] Im AR Kim YH Kim YH Yang WK Kim SH Song KH. Dolichos lablab protects against nonalcoholic fatty liver disease in mice fed high-fat diets. J Med Food. (2017) 20:1222–32. 10.1089/jmf.2017.4036 29090980

[23] Nosworthy MG Dodge ME Bertolo RF Brunton JA. Enterally delivered dipeptides improve small intestinal inflammatory status in a piglet model of intestinal resection. Clin Nutr. (2016) 35:852–8. 10.1016/j.clnu.2015.05.013 26073670

[24] Smith RJ Rao-Bhatia A Kim TH. Signaling and epigenetic mechanisms of intestinal stem cells and progenitors: insight into crypt homeostasis, plasticity, and niches. Wiley Interdiscip Rev Dev Biol. (2017) 6:5. 10.1002/wdev.281 28644919

[25] Zhou W Ramachandran D Mansouri A Dailey MJ. Glucose stimulates intestinal epithelial crypt proliferation by modulating cellular energy metabolism. J Cell Physiol. (2018) 233:3465–75. 10.1002/jcp.26199 28926104

[26] Jiao LF Song ZH Ke YL Xiao K Hu CH Shi B. Cello-oligosaccharide influences intestinal microflora, mucosal architecture and nutrient transport in weaned pigs. Anim Feed Sci Technol. (2014) 195:85–91. 10.1016/j.anifeedsci.2014.05.014

[27] He LQ Niu H Li H Xu ZQ Yao K Li TJ. Effects of dietary L-lysine supplementation on lysine transport by the piglet small intestine *in vitro*. J Anim Sci. (2016) 94:106–10. 10.2527/jas.2015-020726812317

[28] Pedersen KS Kristensen CS Nielsen JP. Demonstration of non-specific colitis and increased crypt depth in colon of weaned pigs with diarrhea. Vet Q. (2012) 32:45–9. 10.1080/01652176.2012.675091 22469034

[29] Sun N Jin Z Li D Yin H Lin S. An exploration of the calcium-binding mode of egg white peptide, Asp-His-Thr-Lys-Glu, and in vitro calcium absorption studies of peptide-calcium complex. J Agric Food Chem. (2017) 65:9782–9. 10.1021/acs.jafc.7b03705 29065689

[30] Huang S Liu H Meng N Li B Wang J. Hypolipidemic and antioxidant effects of malus toringoides (Rehd) hughes leaves in high-fat-diet-induced hyperlipidemic rats. J Med Food. (2017) 20:258–64. 10.1089/jmf.2016.3778 28296591

[31] Smith I Yu J Hurley SL Hanner T. Impact of diet containing grape pomace on growth performance and blood lipid profile of young rats. J Med Food. (2017) 20:550–6. 10.1089/jmf.2016.0117 28384036

[32] Kang S Mullen J Miranda LP Deshpande R. Utilization of tyrosine- and histidine-containing dipeptides to enhance productivity and culture viability. Biotechnol Bioeng. (2012) 109:2286–94. 10.1002/bit.24507 22447498

[33] Su L Zhang Y Cheng YC Lee WM Ye K Hu D. Slc15a1 is involved in the transport of synthetic F5-peptide into the seminiferous epithelium in adult rat testes. Sci Rep. (2015) 5:16271. 10.1038/srep16271 26537751 PMC4633691

[34] Han H Li Y Fang J Liu G Yin J Li T. Gut microbiota and type 1 diabetes. Int J Mol Sci. (2018) 19:995. 10.3390/ijms19040995 29584630 PMC5979537

[35] Kuhn KA Schulz HM Regner EH Severs EL Hendrickson JD Mehta G. Bacteroidales recruit Il-6-producing intraepithelial lymphocytes in the colon to promote barrier integrity. Mucosal Immunol. (2018) 11:357–68. 10.1038/mi.2017.55 28812548 PMC5815964

[36] Zitomersky NL Atkinson BJ Franklin SW Mitchell PD Snapper SB Comstock LE. Characterization of adherent bacteroidales from intestinal biopsies of children and young adults with inflammatory bowel disease. PLoS ONE. (2013) 8:e63686. 10.1371/journal.pone.0063686 23776434 PMC3679120

